# Leveraging artificial intelligence to support surgical oncology multidisciplinary team decision-making: a systematic review

**DOI:** 10.3389/frai.2026.1804004

**Published:** 2026-07-07

**Authors:** Fiona Wu, Rabiya Aseem, Jo Armes, Timothy Rockall, Adam E. Frampton, Farrokh Pakzad

**Affiliations:** 1 Faculty of Health and Medical Sciences, University of Surrey, Guildford, United Kingdom; 2 Minimal Access Therapy Training Unit (MATTU), Royal Surrey NHS Foundation Trust, Guildford, United Kingdom; 3 Department of General Surgery, Royal Surrey NHS Foundation Trust, Guildford, United Kingdom; 4 Division of Cancer, Imperial College London, Hammersmith Hospital Campus, London, United Kingdom

**Keywords:** clinical decision support, decision support, MDT, multidisciplinary team, tumor board

## Abstract

**Introduction:**

Artificial intelligence (AI)–based clinical decision support systems (CDSSs) are increasingly used to support clinicians by providing evidence-based treatment recommendations for multidisciplinary team (MDT) meetings. This systematic review mapped the current landscape of AI-based CDSSs in surgical oncology decision-making and synthesized evidence on their performance and factors influencing effectiveness.

**Methods:**

Cochrane, Ovid MEDLINE, and Embase were searched on February 3, 2025. Studies evaluating AI-based CDSSs for therapeutic decision-making in surgical oncology were included. Methodological quality was assessed using the Critical Appraisal Skills Programme Diagnostic Study Checklist, and data were synthesized narratively.

**Results:**

Fifty-nine studies, encompassing 23,158 patients, were included. CDSSs were classified into five categories: decision tree–based systems, knowledge representation–based systems, Watson for Oncology (WfO), large language models, and other AI-based systems. Concordance with MDT or guideline-based recommendations ranged from 23.2% to 99%. Decision tree– and knowledge-based systems generally demonstrated higher concordance and improved guideline adherence, while WfO performance varied substantially by region and treatment accessibility. Three key themes related to implementation challenges emerged: technical limitations, socioeconomic and healthcare system constraints, and patient- and tumour-specific factors. Reported benefits included improved adherence of MDT decisions to clinical guidelines, enhanced identification of patients eligible for clinical trial enrolment, support for less experienced clinicians, and facilitation of triage for routine cases.

**Discussion:**

AI-based CDSSs show promise in supporting MDT decision-making but remain constrained by challenges related to system maintenance, variability in clinical protocols, therapeutic availability, and patient heterogeneity. Larger prospective studies are needed to evaluate the real-world integration, clinical impact, and patient outcomes of AI-based CDSSs within MDT workflows.

**Systematic review registration:**

https://www.crd.york.ac.uk/PROSPERO/view/CRD42025639227, identifer: CRD42025639227.

## 1 Introduction

Multidisciplinary team (MDT) meetings have become the primary forum for collective decision-making and the standard of care for managing complex cancers (NHS England, 2020). However, the burden on MDTs continues to grow due to rising cancer incidence, improved survival, and increasing case complexity (Winters et al., 2021). Advances in oncology have expanded the therapeutic landscape, with frequent updates to protocols, guidelines, and a growing portfolio of clinical trials. While these developments enhance care, they also challenge clinicians to stay current with rapidly evolving standards.

The value of MDTs is well-established, structured meetings have been shown to improve treatment selection and survival outcomes (Pangarsa et al., 2023; Stephens et al., 2006; Davies et al., 2006; Junor et al., 1994). Conversely, poorly organized meetings may lead to worse outcomes than cases not discussed at all (Lu et al., 2019). Pressures such as excessive caseloads, time constraints, and decision-making fatigue can compromise performance, leading to suboptimal recommendations and increased cognitive strain (Lamb et al., 2011; Soukup et al., 2019).

Artificial intelligence (AI) has emerged as a potential tool to address these challenges. AI-based clinical decision support systems (CDSSs) can process large datasets, summarize evidence, and apply clinical guidelines to support therapeutic decision-making (Jie et al., 2021). These digital tools have shown promise in recommending treatment strategies, prioritizing complex cases, and improving guideline adherence (Oehring et al., 2023; Hendriks et al., 2024; Séroussi et al., 2007). By supporting information processing and decision consistency, AI may help MDTs maintain high-quality outputs despite rising workload and case complexity. This systematic review aimed to characterize the current landscape of AI-based CDSSs in surgical oncology MDTs through a narrative synthesis of their performance and the factors influencing clinical effectiveness.

## 2 Materials and methods

This systematic review followed the Preferred Reporting Items for Systematic Reviews and Meta-Analyses (PRISMA) guidelines. The protocol was registered in PROSPERO (ID: CRD42025639227).

### 2.1 Search strategy

A search was conducted in the Cochrane Library, Ovid MEDLINE, and Embase databases on February 3, 2025. The search strategy combined medical subject headings (MeSH) and free-text terms related to AI, CDSS, MDT meetings, and surgical oncology. Search terms included combinations of: (“artificial intelligence” OR “machine learning” OR “deep learning” OR “natural language processing” OR “large language model” OR “Watson for Oncology” OR “chatbot” OR “ChatGPT”) AND (“clinical decision support” OR “decision support system” OR “clinical decision support system” OR “decision making” OR “computer-assisted decision making”) AND (“tumor board” OR “multidisciplinary team” OR “multidisciplinary cancer team” OR “multidisciplinary meeting” OR “MDM” OR “MDT”) AND (“neoplasm” OR “tumor” OR “carcinoma” OR “cancer” OR “oncology”). No date restrictions were applied. Reference lists of included articles were manually screened to identify additional eligible studies.

### 2.2 Eligibility criteria

Studies were included if they: (1) reported CDSS use in surgical oncology settings; (2) evaluated concordance or predictive accuracy with the MDT, clinical practice or guidelines; (3) peer-reviewed; and (4) published in English. Systems were eligible if they applied symbolic AI or machine learning (ML) to support therapeutic decision-making across multiple patient or tumor variables. Development and validation studies were also included. Studies were excluded if they did not evaluate CDSS concordance or accuracy, focused solely on clinician and/or patient acceptance, behavioral influence, prognostication, or trial eligibility. Reviews, case reports, editorials, commentaries, and conference abstracts lacking detailed data were also excluded.

### 2.3 Study selection

**Figure 1:**
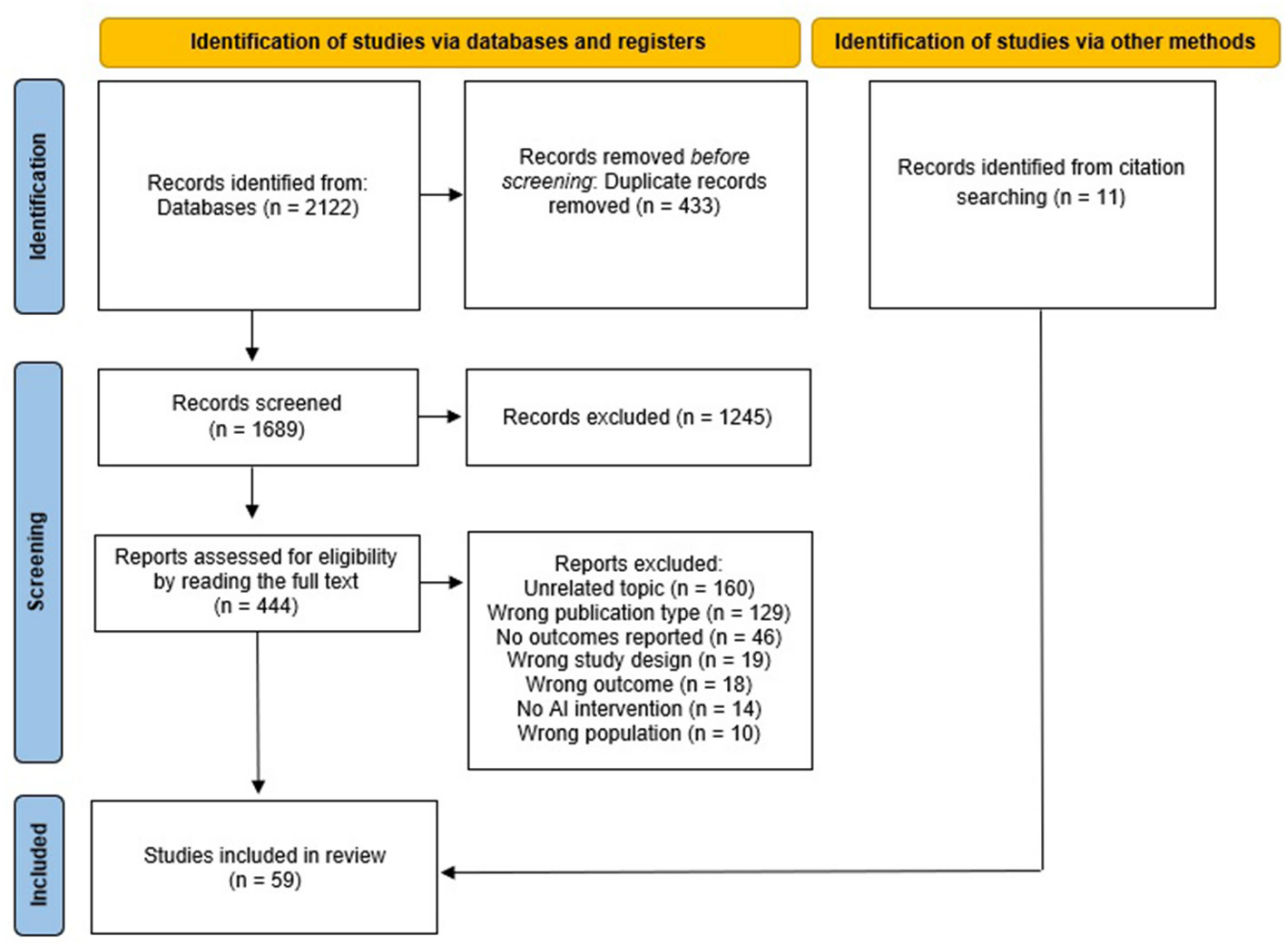
PRISMA flow diagram.

Search results were imported into Rayyan for screening. After duplicates were removed, two reviewers (FW and RA) independently screened titles and abstracts. Full texts were reviewed for potentially eligible studies. Disagreements were resolved through discussion and consensus. Reference lists of included articles were manually searched for additional studies. The process is illustrated in the PRISMA flow diagram (Figure 1).

### 2.4 Quality assessment

Methodological quality was assessed using the Critical Appraisal Skills Programme (CASP) Diagnostic Study Checklist, this tool was selected due to the conceptual similarity between CDSS performance and diagnostic accuracy assessment. The checklist assesses study quality across validity, results, and applicability domains, with each study rated as low, moderate, or high quality.

### 2.5 Data extraction

Data was extracted using a standardized form capturing: (1) study details (author, year, country); (2) design and population; (3) CDSS type; (4) performance outcomes; and (5) variables influencing performance. The primary outcome was concordance, the proportion of agreement between CDSS-generated recommendations and the MDT or guideline-based decisions, reported as a percentage. Where available, additional effect measures such as odds ratios (ORs), hazard ratios (HRs), accuracy, and area under the receiver operating characteristic curve (AUC) were extracted to assess predictive performance. For the narrative synthesis, descriptive summaries were used to present contextual influences and implementation factors on system performance.

### 2.6 Synthesis methods

Given the methodological and clinical heterogeneity among studies, meta-analysis was not feasible. Studies were categorized by CDSS type and tumor groups, and are presented in the results tables. A narrative synthesis was conducted to integrate quantitative findings and to examine influential variables and implementation factors. No formal sensitivity analyses were performed. Rather than pooling accuracy estimates, this review aimed to provide a comprehensive overview of system performance, limitations, and practical applications in surgical oncology. Robustness was supported by cross-comparison of findings across study designs, tumor groups, and geographical regions to identify consistent patterns and potential outliers.

## 3 Results

### 3.1 Study selection

Two thousand one hundred twenty-two records were identified through the initial search. Eleven studies were identified through citation tracking. Fifty-nine studies met the inclusion criteria and were included in the final review. The study selection process is outlined in the PRISMA flow diagram (Figure 1).

### 3.2 Study characteristics

Twenty-three thousand, one hundred fifty-eight patients were evaluated across a range of tumor types, including breast, colorectal, lung, oesophagogastric (OG), head and neck (H&N), urological, skin, gynecological, thyroid, hepato-pancreato-biliary (HPB), and glioma. Four studies included patients from mixed tumor groups (Table 1). “Reference decision” is used to denote the standard against which the CDSS-generated recommendations were compared (e.g., MDT decisions, clinical guidelines, clinical practice). The median sample size was 175 patients (interquartile range: 61–362). System approaches were broadly classified into five categories: (1) decision tree–based systems; (2) knowledge representation–based systems; (3) Watson for Oncology (WfO); (4) large language models (LLMs); and (5) other AI-based systems.

**Table 1:** Identified studies for systematic review.

References	Country	Study design	Sample size	Tumor group	Decision support	CDSS type	Reference decision
Séroussi et al. (2007)	France	Before/after implementation	316	Breast	OncoDoc2	Decision tree	MDT
O'Reilly et al. (2008)	UK	Retrospective	98	Colorectal	OncoSurge	Decision tree	MDT
Patkar et al. (2012)	UK	Observational	1,056	Breast	MATE	Knowledge representation	MDT
Séroussi et al. (2013)	France	Observational	1,624	Breast	OncoDoc2	Decision tree	MDT
Sesen et al. (2014)	UK	Retrospective	4,020	Lung	Lung Cancer Assistant	Rule-based and Bayesian network	Clinical practice
Eccher et al. (2014)	Italy	Validation	61	Breast	OncoCure	Knowledge representation	MDT
Lin et al. (2016)	Australia	Retrospective	1,065	Breast	–	ML	MDT
Somashekhar et al. (2018)	India	Retrospective	638	Breast	WfO	NLP, ML and knowledge based	MDT
Lee et al. (2018)	South Korea	Retrospective	656	Colorectal	WfO	NLP, ML and knowledge based	MDT
Kim et al. (2018)	South Korea	Retrospective	95	Breast	WfO	NLP, ML and knowledge based	Clinical practice
Liu et al. (2018)	China	Retrospective	149	Lung	WfO	NLP, ML and knowledge based	MDT
Zhou et al. (2019)	China	Retrospective	362	Breast, lung, gastric, colon, rectal, cervical, ovarian	WfO	NLP, ML and knowledge based	MDT
Choi et al. (2019)	South Korea	Retrospective	65	Gastric	WfO	NLP, ML and knowledge based	MDT
Kim et al. (2019a)	South Korea	Retrospective	69	Colorectal	WfO	NLP, ML and knowledge based	MDT
Pan et al. (2019)	China	Retrospective	1,301	Breast	WfO	NLP, ML and knowledge based	Clinical practice
Kim et al. (2019b)	South Korea	Retrospective	207	Thyroid	WfO	NLP, ML and knowledge based	Clinical practice
McNamara et al. (2019)	USA	Observational	88	Breast	WfO	NLP, ML and knowledge based	Clinical expertise
Xu et al. (2019)	China	Observational	1,977	Breast	WfO	NLP, ML and knowledge based	Clinical practice
Zhang et al. (2020)	China	Retrospective	243	HCC	WfO	NLP, ML and knowledge based	Clinical practice
Yao et al. (2020)	China	Retrospective	165	Lung	WfO	NLP, ML and knowledge based	Physicians
You et al. (2020)	China	Retrospective	310	Lung	WfO	NLP, ML and knowledge based	Clinical practice
Tian et al. (2020)	China	Retrospective	235	Gastric	WfO	NLP, ML and knowledge based	MDT
Zhao et al. (2020)	China	Retrospective	302	Breast	WfO	NLP, ML and knowledge based	MDT
Kim et al. (2020)	South Korea	Retrospective	405	Lung	WfO	NLP, ML and knowledge based	MDT
Mao et al. (2020)	China	Retrospective	175	Colorectal	WfO	NLP, ML and knowledge based	MDT
Zou et al. (2020)	China	Retrospective	246	Cervical	WfO	NLP, ML and knowledge based	Clinical practice
Aikemu et al. (2021)	China	Observational	250	Colorectal	WfO	NLP, ML and knowledge based	MDT
Yu et al. (2021)	South Korea	Retrospective	201	Prostate	WfO	NLP, ML and knowledge based	Clinical practice
Suwanvecho et al. (2021)	Thailand	Retrospective	313	Breast, lung, colon, rectal	WfO	NLP, ML and knowledge based	Clinical practice
Yun et al. (2021)	South Korea	Retrospective	50	Thyroid	WfO	NLP, ML and knowledge based	Clinical practice
Andrew et al. (2022)	UK	Retrospective	304	Skin (BCC)	–	Decision tree	MDT
Keikes et al. (2021)	Netherlands	Validation	109	Colorectal	Oncoguide	Decision tree	MDT
Ebben et al. (2022)	Netherlands	Multi-center observational	355	Breast, colorectal, prostate	Oncoguide	Decision tree	MDT
Griewing et al. (2023)	Germany	Observational	20	Breast	ChatGPT-3.5	LLM	MDT
Hikal et al. (2023)	Germany	Validation	92	Laryngeal	–	Bayesian network	Clinical practice
Ural et al. (2023)	Germany	Retrospective	1,873	Prostate	EasyOncology	Decision tree	MDT
Park et al. (2023)	South Korea	Retrospective	55	Prostate, bladder, kidney	WfO	NLP, ML and knowledge based	Clinical practice
Liu et al. (2023)	China	Retrospective	463	Breast	WfO	NLP, ML and knowledge based	Clinical practice
Haemmerli et al. (2023)	Switzerland	Retrospective	10	Glioma	ChatGPT-3.5	LLM	MDT
Thavanesan et al. (2023)	UK	Retrospective	399	Esophageal	–	ML	Clinical practice
Lukac et al. (2023)	Germany	Observational	10	Breast	ChatGPT-3.5	LLM	MDT
Choo et al. (2024)	South Korea	Observational	30	Colorectal	ChatGPT	LLM	MDT
Park and Chae 2024)	South Korea	Retrospective	322	Gastric	WfO	NLP, ML and knowledge based	MDT
Daye et al. (2024)	USA	Retrospective	140	HCC	-	ML	MDT
Xu et al. (2024)	China	Retrospective	537	Breast	CSCO AI	Knowledge graph	Clinical practice
Ali et al. (2024)	UK	Retrospective	893	Skin (BCC)	vSMDT	NLP	MDT
Aghamaliyev et al. (2024)	Germany	Retrospective	93	Gastric, esophageal, PDAC, CCC, and HCC	ChatGPT-3.5	LLM	MDT
Griewing et al. (2024a)	Germany	Observational	20	Breast	ChatGPT-3.5, ChatGPT-4, Llama-2, and Bard	LLM	MDT
Griewing et al. (2024b)	Germany	Observational	20	Breast	ChatGPT-3.5, ChatGPT-4, BC-SLM	Large and small language models	MDT
Schmidl et al. (2024a)	Germany	Observational	20	H&N SCC	ChatGPT-3.5 and 4.0	LLM	MDT
Schmidl et al. (2024b)	Germany	Observational	50	H&N SCC	ChatGPT-4.0 and Claude-3-Opus	LLM	MDT
Schmidl et al. (2024c)	Germany	Observational	100	H&N SCC	ChatGPT-4o and 4.0	LLM	MDT
Ebner et al. (2024)	Germany	Retrospective	10	Cervical	ChatGPT-3.5	LLM	MDT
Li et al. (2024)	China	Retrospective	213	Gastric	GC-CDSS	Knowledge graph	MDT
Hoier et al. (2024)	Germany	Retrospective	111	Malignant melanoma	EasyOncology	Decision tree	MDT
Horesh et al. (2025)	USA	Observational	15	Colorectal	ChatGPT-3.5	LLM	MDT
Kaiser et al. (2025)	Switzerland	Retrospective	171	Prostate	ChatGPT-4 and Claude-3-Opus	LLM	MDT
Buhr et al. (2025)	Germany	Observational	25	ENT	ChatGPT-4o and Llama-3	LLM	MDT
Zabaleta et al. (2025)	Spain	Retrospective	52	Lung	ChatGPT-3.5	LLM	MDT

CDSS, clinical decision support system; MDT, multidisciplinary team; ML, machine learning; WfO, Watson for Oncology; NLP, natural language processing; HCC, hepatocellular carcinoma; BCC, basal cell carcinoma; LLM, large language model; PDAC, pancreatic ductal adenocarcinoma; CCC, cholangiocarcinoma; H&N SCC„ head and neck squamous cell carcinoma; ENT, ear, nose and throat.

### 3.3 Performance by type of clinical decision support system

#### 3.3.1 Decision tree

Eight studies evaluated CDSSs using decision tree algorithms derived from established clinical practice guidelines (Table 2). These systems operationalized guideline logic by mapping patient and tumor characteristics onto predefined decision pathways and were applied across breast, colorectal, prostate, and skin cancers. Overall, decision tree-based CDSSs demonstrated high concordance with MDT recommendations in guideline-aligned clinical scenarios.

**Table 2:** Overview of decision tree-based systems.

References	Sample size	Tumor group	Decision support	Outcome measurement	Performance of decision support	Notable findings	Quality assessment
Séroussi et al. (2007)	316	Breast	OncoDoc2	Binary concordance	93.4%	- Guideline adherence before/after MDT implementation (79.2%/93.4%)	Moderate (55%)
Séroussi et al. (2013)	1,624	Breast	OncoDoc2	Exact concordance	91.7%	–	High (77%)
O'Reilly et al. (2008)	98	Colorectal	OncoSurge	Binary concordance	94.9%	–	Moderate (68%)
Keikes et al. (2021)	109	Colorectal	Oncoguide	Binary concordance	81%	- No guideline recommendation was available in 92% of discordant cases	Moderate (55%)
Ebben et al. (2022)	118	Breast	Oncoguide	Exact and partial concordance	- Total 94.1% - Exact 85.3% - Partial 8.8%	MDT reasons for discordant cases: - Specific tumor characteristics (*n* = 3) - Comorbidity (*n* = 2) - Clinical trial inclusion (*n* = 1)	Moderate (64%)
	111	Colorectal			- Total 94.5% - Exact 88.9% - Partial 5.6%	MDT reasons for discordant cases: - Clinical trial inclusion (*n* = 10) - Specific tumor characteristics (*n* = 4) - Comorbidity (*n* = 3) - Age (*n* = 1) - Current clinical guideline outdated (*n* = 1)	
	126	Prostate			- Total 88.4% - Exact 78.8% - Partial 9.6%	MDT reasons for discordance: - Clinical trial inclusion (*n* = 10) - Specific tumor characteristics (*n* = 4) - Comorbidity (*n* = 3) - Age (*n* = 1) - Current clinical guideline outdated (*n* = 1)	
Ural et al. (2023)	1,873	Prostate	EasyOncology	Tiered recommendation concordance	- Total 99% - Recommended 93.6% - For consideration 5.4%	Variables influencing concordance: - Age OR 0.955 (95% CI: 0.898–1.015), *P* 0.141 - PSA OR 1 (95% CI: 1.000–1.001), *P* 0.985 - Stage (≥3) OR 2.210 (95% CI: 0.832–5.870), *P* 0.112	High (91%)
Hoier et al. (2024)	111	Skin (malignant melanoma)		Exact and partial concordance	- Total 97% - Exact 78% - Partial 19%	Reasons for discordance: - Diagnostic uncertainty (*n =* 2) - Advanced patient age (*n =* 1)	High (82%)
Andrew et al. (2022)	304	Skin (BCC)	ML algorithm	Binary concordance	45.1%	Variables associated with MMS: - Younger (66.8 vs. 74.9 years; *P* < 0.0001) - Less likely to be anticoagulated (15 vs. 35.58%; *P* < 0.0001) - Less likely to have a permanent pacemaker *in situ* (1.5 vs. 8.65%; *P* 0.004)	High (95%)

MDT, multidisciplinary; OR, odds ratio; CI, confidence interval; BCC, basal cell carcinoma; ML, machine learning.

Among the earliest systems evaluated, OncoDoc2 was assessed in French breast cancer MDTs and showed a substantial improvement in guideline adherence following implementation, increasing from 79.2% prior to integration to over 93% after deployment (Séroussi et al., 2007). This high level of agreement was sustained in subsequent validation studies involving larger cohorts, with concordance consistently exceeding 90% (Séroussi et al., 2007, 2013). Comparable performance was observed with Oncoguide, which achieved 81% concordance in colorectal cancer (Keikes et al., 2021). Notably, most discordant cases reflected clinical scenarios not addressed by existing guidelines rather than disagreement with CDSS-based recommendations.

This pattern was reinforced in multi-center evaluations of Oncoguide across breast, colorectal, and prostate cancer cohorts, where concordance rates exceeded 88% (Ebben et al., 2022). Reported reasons for discordance were predominantly related to patient-specific factors beyond guideline scope, including comorbidities, advanced age, tumor-specific characteristics, and enrolment in clinical trials. In addition, a proportion of cases could not be assessed because of incomplete clinical information available at the time of MDT discussion.

Very high concordance was reported with EasyOncology, which achieved 99% agreement with MDT decisions in prostate cancer (Séroussi et al., 2013). Similarly, in malignant melanoma, concordance reached 97%, with discordance primarily attributed to diagnostic uncertainty or advanced patient age (Sesen et al., 2014). In contrast, a decision tree model applied to basal cell carcinoma demonstrated substantially lower concordance when used to triage suitability for Mohs micrographic surgery, underscoring limitations in capturing nuanced anatomical considerations and patient-specific factors (Andrew et al., 2022). Collectively, these findings indicate that decision tree-based CDSSs closely replicate MDT decision-making when clinical scenarios are well-defined and comprehensively covered by guidelines.

#### 3.3.2 Knowledge representation

Six studies evaluated CDSSs employing knowledge representation approaches, including rule-based systems, knowledge graphs, and probabilistic models such as Bayesian networks (Table 3). These systems encoded clinical knowledge derived from guidelines, expert consensus, or national datasets to generate treatment recommendations and were applied across breast, gastric, laryngeal, and lung cancers. Overall, knowledge representation-based CDSSs demonstrated moderate to high concordance with MDT decisions.

**Table 3:** Overview of knowledge representation-based systems.

References	Sample size	Tumor group	Decision support	Outcome measurement	Performance of decision support	Notable findings	Quality assessment
Patkar et al. (2012)	1,056	Breast	MATE	Binary concordance	93.2%	- MATE identified 61% more patients eligible for clinical trial recruitment - 95.8% of feedback respondents agreed that MATE has a valuable role in cancer MDTs	Moderate (68%)
Eccher et al. (2014)	61	Breast	OncoCure	Binary concordance	85.2%	–	Moderate (50%)
Xu et al. (2024)	537	Breast	CSCO AI	Binary concordance	80.4%	Variables influencing concordance: - Age ≤ 44 years OR 2 (95% CI: 1.027–4.179), *P* 0.042 - Age 44–55 years OR 2 (95% CI: 1.140–3.402), *P* 0.015 - Post-menopausal status OR 2 (95% CI: 1.198–3.475), *P* 0.009 - Triple negative molecular subtype OR 2.1 (95% CI: 0.944–4.780), *P* 0.069	High (86%)
Li et al. (2024)	213	Gastric	GC-CDSS	Binary concordance	92.96%	–	Moderate (68%)
Sesen et al. (2014)	4,020	Lung	Lung cancer assistant	Exact and partial concordance	Guideline-based: - Exact 57% - Partial 79% Probabilistic: - Exact 27% - Partial 76%	–	Moderate (73%)
Hikal et al. (2023)	460	Laryngeal	Bayesian network	Binary concordance	91%	–	Moderate (64%)

MDT, multidisciplinary; OR, odds ratio; CI, confidence interval.

In breast cancer, several systems showed strong agreement with MDT recommendations. The MATE system achieved 93.2% concordance in a cohort of over 1,000 patients and additionally identified a higher proportion of patients eligible for clinical trial enrolment compared with MDT review alone (Patkar et al., 2012). In a separate Chinese cohort, CSCO AI, a CDSS that integrates clinical guidelines, patient data, and expert consensus within a knowledge graph framework, demonstrated 80.4% concordance, with agreement varying according to patient age, menopausal status, and tumor molecular subtype (Xu et al., 2024). Similarly high concordance was reported in gastrointestinal and head and neck malignancies. GC-CDSS, which employs a comparable knowledge-based approach, achieved nearly 93% concordance in gastric cancer, while a Bayesian network model in laryngeal cancer demonstrated 91% agreement with expert recommendations (Zhou et al., 2019; Li et al., 2024). In lung cancer, performance varied by system component. A hybrid CDSS combining a guideline-based rule engine with a probabilistic Bayesian network achieved higher concordance using the rule-based component than the probabilistic component, although partial concordance remained high across both approaches (Sesen et al., 2014). Across tumor types, these systems performed best in guideline-aligned scenarios, with variability reflecting differences in knowledge sources and system architecture.

#### 3.3.3 Watson for Oncology

The largest body of evidence for a single system related to IBM WfO, which was evaluated in 26 studies conducted across China, South Korea, Thailand, India, and the United States (Table 4). WfO is a knowledge-based CDSS that integrates natural language processing with limited ML and an expert-curated knowledge base derived primarily from National Comprehensive Cancer Network (NCCN) guidelines and clinical expertise from Memorial Sloan Kettering Cancer Center. The system generates ranked treatment options categorized as “recommended,” “for consideration,” or “not recommended.” Across tumor types, concordance between WfO recommendations and MDT decisions varied widely, ranging from 23.2% to 95.83% (Kim et al., 2018; Zhou et al., 2019). Lower concordance was generally observed in advanced disease, older patient populations, and healthcare settings with limited access to recommended therapies. Several studies identified demographic, clinical, and system-level factors as statistically significant predictors of concordance.

**Table 4:** Overview of Watson for Oncology.

References	Sample size	Tumor group	Outcome measurement	Performance of decision support	Notable findings	Quality assessment
Somashekhar et al. (2018)	638	Breast	Tiered recommendation concordance	- Total 93% - Recommended 62% - For consideration 31%	Variables influencing concordance: - Age 65–74 years OR 0.07 (95% CI: 0.02–0.30), *P* < 0.001 - Age 75+ years OR 0.007 (95% CI: 0.001–0.04), *P* < 0.001 - Stage II OR 16.07 (95% CI: 1.74–148.34), *P* 0.01 - Stage III OR 8.53 (95% CI: 1.78–40.77), *P* 0.01 - Stage IV OR 23.24 (95% CI: 2.32–233.09), *P* 0.01 - Stage IV Triple OR 0.02 (95% CI: 0.001–0.76), *P* 0.04	High (86%)
Pan et al. (2019)	1,301	Breast	Tiered recommendation concordance	- Total 69.4% - Recommended 25.1% - For consideration 44.3%	Variables influencing concordance in adjuvant chemotherapy group: - Age 70+ years OR 0.33 (95% CI: 0.14–0.78), *P* 0.012 - Stage II OR 2.12 (95% CI: 1.57–2.88), *P* < 0.001 - Stage III OR 13.87 (95% CI: 7.66–25.11), *P* < 0.001 - Triple negative OR 5.95 (95% CI: 2.25–15.75), *P* < 0.001	High (86%)
Xu et al. (2019)	1,977	Breast	Binary concordance	56%	Clinicians' reasons for discordance: - Clinical judgement at variance with WfO (48%) - WfO not consistent with NCCN (33%) - Treatment not available in WfO (8%) - Treatment not available in China (11%)	High (86%)
Kim et al. (2018)	95	Breast	Binary concordance	23.2%	Without/with GEA: - Sensitivity 92%/100% - Specificity 21.4%/80% - PPV 29.5%/61% - NPV 88.2%/100%	Moderate (50%)
McNamara et al. (2019)	88	Breast	Tiered recommendation concordance	- Total 87.9% - Recommend 78.5% - For consideration 9.4%	Concordance with WfO recommendations by oncologist subspeciality (without/with): - Breast cancer expert (80.8%/80.8%) - Solid tumor (66.7%/86.7%) - Hematological (60.7%/88.5%) - Breast cancer novices (62.5%/87.9%)	Moderate (59%)
Zhao et al. (2020)	200	Breast (adjuvant)	Tiered recommendation concordance	- Total 77% - Recommended 75.5% - For consideration 19.5%	Reasons for discordance: - Patients refused recommended treatment due to no reimbursement (19.6%) - Differences in chemotherapy decision-making (69.6%) - Patient refusal (8.7%) - Patient could not tolerate treatment (2.1%)	High (82%)
	102	Breast (metastatic)		- Total 27.5% - Recommended 17.7% - For consideration 9.8%	Reasons for discordance: - Unavailability of CDK4/6 inhibitors in China (40.5%) - Patients refused recommended treatment due to no reimbursement (5.5%) - WfO recommended single-agent chemotherapy, whilst the MDT recommended combination therapy (43.2%) - Differences in other chemotherapy decision-making (10.8%)	
Liu et al. (2023)	263	Breast (adjuvant)	Binary concordance	80.2%	Variables influencing concordance: - Menopausal status OR 2.89 (95% CI: 1.260–6.63), *P* 0.012 - Histological grade OR 0.22 (95% CI: 0.061–0.781), *P* 0.019 - Stage OR 0.22 (95% CI: 0.050–0.943), *P* 0.042	Moderate (68%)
	200	Breast (advanced)		50.5%	–	
Lee et al. (2018)	656	Colorectal	Tiered recommendation concordance	- Total 65.8% - Recommended 48.9% - For consideration 16.9%	- Guideline adherence before/after MDT implementation 38.9%/47.4% (*P* 0.402) - Concordance before/after implementation of biologic agent reimbursement 64.2%/20.3% (*P* 0.003)	High (82%)
Kim et al. (2019a)	69	Colorectal	Tiered recommendation concordance	- Total 87% - Recommended 46.4% - For consideration 40.6%	- Cancer stage (II, IIIC, or IV) significantly influenced concordance OR 0.092 (95% CI: 0.030–0.282), *P* < 0.001	Moderate (59%)
Mao et al. (2020)	175	Colorectal	Tiered recommendation concordance	- Total 66.9% - Recommended 44% - For consideration 22.9%	- OS was better in “recommended”, than “not recommended” group (*P* 0.008) - Non-concordance is an independent risk factor for OS; HR 2.784 (95% CI:1.264–6.135) Variables influencing concordance: - Left colon cancer OR 2.195 (95% CI 0.911–5.291), *P* 0.080 - Rectal cancer OR 2.5 (95% CI: 1.061–5.881), *P* 0.036 - Stage III OR 0.187 (95% CI: 0.050–0.707), *P* 0.013 - Stage IV OR 0.127 (95% CI: 0.031–0.711), *P* 0.017	High (77%)
Aikemu et al. (2021)	250	Colorectal	Tiered recommendation concordance	- Total 91% - Recommended 80.8% - For consideration 10%	- OS was better in concordant, than non-concordant group (*P* 0.0049)	High (82%)
Liu et al. (2018)	149	Lung	Tiered recommendation concordance	- Total 65.8% - Recommended 42.3% - For consideration 23.5%	Variables influencing concordance: - Pathological type OR 0.09 (95% CI: 0.02–0.45), *P* 0.004 - Stage III OR 3.51 (95% CI: 1.03–12.0), *P* 0.05 - Stage IV OR 9.5 (95% CI: 3.4–26.1), *P* < 0.001 - Non measured EGFR gene mutation OR 0.32 (95% CI: 0.12–0.86), *P* 0.02	High (82%)
Yao et al. (2020)	165	Lung	Binary concordance	73.3%	Variables influencing concordance: - Stage OR 4.095 (95% CI: 1.321–12.694), *P* 0.019 Variables with no significant effect on concordance: gender, performance status, smoking or pathology	High (91%)
You et al. (2020)	310	Lung	Tiered recommendation concordance	- Total 85.16% - Recommended 34.52% - For consideration 50.64%	Variables influencing concordance: - Gene mutation OR 25.979 (95% CI: 4.135–163.218), *P* 0.001	High (82%)
Kim et al. (2020)	405	Lung	Binary concordance (“recommended” treatment only)	92.4%	–	High (82%)
Choi et al. (2019)	65	Gastric	Tiered recommendation concordance	- Recommended 41.5% - For consideration 87.7%	- Stage IV or recurrent disease was significantly more likely to be concordant compared with stage II/III OR 1.6 (95% CI: 0.3–9.4), *P* 0.02	High (77%)
Tian et al. (2020)	235	Gastric	Tiered recommendation concordance	- Total 54.5% - Recommended 43% - For consideration 11.5%	- The average survival time was 30.0 months for concordant patients compared to 16.4 months for discordant patients (*P* < 0.001) - Concordance is an independent prognostic factor for survival HR 0.31 (95% CI: 0.187–0.521), *P* < 0.001	High (91%)
Park and Chae 2024)	322	Gastric	Tiered recommendation concordance	- Total 86.96% - Recommended 72.98% - For consideration 13.98%	Variables influencing concordance: - Age ≥ 80 years OR 0.175 (95% CI: 0.069–0.441), *P* 0.000 - Male sex OR 2.006 (95% CI: 0.916, 4.391), *P* 0.082 - Performance status 1 OR 0.203 (95% CI: 0.072–0.574), *P* 0.003 - Performance status 2 OR 0.191 (95% CI: 0.057–0.639), *P* 0.007 - Performance status 3 OR 0.089 (95% CI: 0.026–0.301), *P* 0.000 - Cancer stage IV OR 0.017 (95% CI: 0.005–0.055), *P* 0.017	Moderate (73%)
Yu et al. (2021)	201	Prostate	Tiered recommendation concordance	- Total 73.6% - Recommended 53.2% - For consideration 20.4%	Variables influencing concordance: - Poor performance status (≥1) OR 14.75 (95% CI: 6.56–33.16), *P* 0.001 - Age ≥ 75 years OR 2.47 (95% CI: 1.12–5.44), *P* 0.026	High (82%)
Park et al. (2023)	48	Prostate	Binary concordance (“recommended” treatment only)	Overall 92.7%	Reasons for discordance: - High cost of enzalutamide (n = 1) - No option in WfO (n = 3)	Moderate (55%)
	5	Bladder				
	2	Kidney				
Zhang et al. (2020)	243	HCC	Tiered recommendation concordance	- Total 72% - Recommended 67% - For consideration 5%	Variables influencing concordance: - Multiple tumors OR 0.309 (95% CI: 0.140–0.683), *P* 0.004 - Major hepatectomy OR 0.384 (95% CI: 0.201–0.735), *P* 0.004 - Portal hypertension OR 0.376 (95% CI: 0.162–0.870), *P* 0.022	High (91%)
Zou et al. (2020)	246	Cervical	Tiered recommendation concordance	- Total 72.8% - Recommended 41.5% - For consideration 31.3%	Variables influencing concordance: - Age ≥ 65 years of age OR 0.08 (95% CI: 0.03–0.28), *P* 0.032 - Rural registration OR 0.64 (95% CI: 0.427–0.946), *P* 0.025 - Poor performance status OR 0.29 (95% CI: 0.083–1.058), *P* 0.048 - Stage II OR 2.08 (95% CI: 1.002–4.325), *P* 0.046 - Stage III OR 2.09 (95% CI: 1.001–4.381), *P* 0.047 - Stage IV OR 0.19 (95% CI: 0.038–0.91), *P* 0.025	High (91%)
Kim et al. (2019b)	207	Thyroid	Tiered recommendation concordance	- Total 77% - Recommended 77% - For consideration <1%	Variables influencing concordance: - Intermediate risk OR 0.16 (95% CI: 0.05–0.43), *P* < 0.001 - Stage III OR 0.35 (95% CI: 0.16–0.71), *P* 0.004	Moderate (73%)
Yun et al. (2021)	50	Thyroid	Binary concordance (“recommended” treatment only)	48%	Concordance by stage: - Stage I 52.4% - Stage II 50% - Stage III 16.7%	Moderate (50%)
Zhou et al. (2019)	120	Breast	Tiered recommendation concordance	64.2%	- No significant differences were observed amongst different stages and molecular subtypes	Moderate (50%)
	113	Lung		81.3%		
	42	Gastric		11.9%	–	
	25	Colon		40%	–	
	24	Rectal		74%	–	
	14	Cervical		50%	–	
	24	Ovarian		95.83%	–	
Suwanvecho et al. (2021)	126	Breast	Tiered recommendation concordance	59.5%	–	Moderate (55%)
	88	Lung		68.2%	–	
	70	Colon		84.3%	–	
	29	Rectal		86.2%	–	

OR, odds ratio; CI, confidence interval; WfO, Watson for Oncology; NCCN, National Cancer Comprehensive Network; GEA, gene expression assay; PPV, positive predictive values; NPV, negative predictive values; MDT, multidisciplinary team; OS, overall survival; HR, hazard ratio; HCC, hepatocellular carcinoma.

Breast cancer was the most extensively evaluated tumor type, with reported concordance ranging from 23.2% to 93% (Somashekhar et al., 2018; Kim et al., 2018). Increasing age emerged as a consistent predictor of discordance, with patients aged ≥70–75 years demonstrating significantly lower agreement across multiple studies (Somashekhar et al., 2018; Pan et al., 2019). Disease stage also influenced concordance, with higher alignment observed in stage II–III disease and reduced agreement in metastatic settings (Somashekhar et al., 2018; Pan et al., 2019; Liu et al., 2023). In Zhao et al., discordance was primarily attributable to differences in chemotherapy selection and patient refusal of recommended treatments due to reimbursement constraints (Zhao et al., 2020). Concordance was particularly low in metastatic breast cancer, driven by limited access to CDK4/6 inhibitors, which had not yet been approved for use in China, and divergence between WfO-recommended single-agent regimens and MDT-preferred combination therapies.

In colorectal cancer, concordance ranged from 65.8% to 91%, with higher agreement in early-stage disease and declining concordance in stage III and IV cancer (Liu et al., 2018; Kim et al., 2019a; Mao et al., 2020; Aikemu et al., 2021). Two studies reported significantly improved overall survival among patients receiving WfO-concordant treatment, with non-concordance identified as an independent adverse prognostic factor (Mao et al., 2020; Aikemu et al., 2021). However, advanced disease stage was associated with both lower concordance and poorer survival, suggesting potential confounding (Mao et al., 2020). System-level factors were again influential. In South Korea, changes in reimbursement policies for biologic agents substantially affected concordance (Lee et al., 2018). Prior to reimbursement, concordance in metastatic disease was 64.2%, but following policy changes, WfO's continued prioritization of non-biologic regimens resulted in a marked reduction in concordance to 20.3%.

Lung cancer studies reported concordance rates ranging from 65.8% and 92.4% (Liu et al., 2018; Kim et al., 2020). The presence of gene mutations, particularly EGFR, was associated with significantly higher concordance (Liu et al., 2018; You et al., 2020). Disease stage also influenced performance, with advanced-stage disease generally demonstrating higher agreement, in contrast to patterns observed in other tumor groups (Liu et al., 2018; Yao et al., 2020).

In gastric cancer, concordance ranged from 54.5% to 87.7%, with stage IV or recurrent disease not consistently predicting agreement (Choi et al., 2019; Tian et al., 2020). Choi et al. (2019) identified financial constraints and local practice patterns as prominent contributors to discordance, including lack of insurance coverage for recommended regimens, regional differences in chemotherapy protocols, and variation in preferred treatment sequencing. Consistent with findings from colorectal cancer studies evaluating survival outcomes, one gastric cancer study reported significantly longer mean survival among patients treated concordantly with WfO recommendations, identifying concordance as an independent prognostic factor (Tian et al., 2020).

Across other tumor types, similar trends were observed. In prostate cancer, concordance was generally high (73.6%−92.7%) but declined significantly among older patients and those with poor performance status (Schmidl et al., 2024b,c). Cervical cancer studies reported overall concordance ranging from 50% to 72.8%, with lower agreement observed in older patients, those with poorer performance status, and individuals from rural settings (Zhou et al., 2019; Horesh et al., 2025). In thyroid cancer, discordance was associated with intermediate-risk, stage III disease and differences in surgical extent, as WfO recommendations did not consistently reflect additional clinicopathological details or family history considered by clinicians (Buhr et al., 2025; Zabaleta et al., 2025).

Overall, WfO demonstrated highly variable performance across tumor types and healthcare systems. Concordance was reduced when clinical decision-making was influenced by patient-specific factors, local practice patterns, or treatment availability that had not yet been incorporated into the WfO knowledge base. These findings highlight both the utility of WfO in standardized clinical scenarios and the limitations of guideline-centric CDSSs in more complex, personalized, or resource-constrained contexts.

#### 3.3.4 Large language models

Fifteen studies evaluated LLMs, primarily different iterations of ChatGPT, as CDSSs across a range of tumor types (Table 5). Performance varied widely depending on the model version, tumor context, and evaluation methodology. Early assessments of ChatGPT-3.5 reported limited concordance with MDT decisions. For example, Lukac et al. observed only 16% agreement in breast cancer cases, largely reflecting the model's inability to incorporate patient-specific nuances, while Griewing et al. reported 50% concordance across broader breast cancer scenarios, with the lowest performance in complex cases (Griewing et al., 2023; Lukac et al., 2023; Griewing et al., 2024a). In contrast, Choo et al. reported substantially higher concordance (86.7%) in advanced and recurrent colorectal cancer using an unspecified version of ChatGPT (Choo et al., 2024).

**Table 5:** Overview of large language models.

References	Sample size	Tumor group	Decision support	Outcome measurement	Performance of decision support	Notable findings	Quality assessment
Lukac et al. (2023)	10	Breast	ChatGPT-3.5	Likert-scale concordance	16.05%	–	Low (36%)
Griewing et al. (2023)	20	Breast	ChatGPT-3.5	Binary concordance	50%	–	Moderate (64%)
Griewing et al. (2024a)	20	Breast	ChatGPT-3.5	Binary concordance	35%−50%	–	Moderate (64%)
			ChatGPT-4		60%	–	
			Llama-2		30%	–	
			Bard		20%	–	
Griewing et al. (2024b)	20	Breast	ChatGPT-3.5	Binary concordance	83%	–	Moderate (73%)
			ChatGPT-4		90%	–	
			BC-SLM		86%	–	
Choo et al. (2024)	30	Colorectal	ChatGPT	Likert-scale concordance	86.7%	–	Moderate (64%)
Horesh et al. (2025)	15	Colorectal	ChatGPT-3.5	Likert-scale concordance	4.08	–	Low (45%)
Zabaleta et al. (2025)	52	Lung	ChatGPT-3.5	Binary concordance	76%	–	Moderate (55%)
Aghamaliyev et al. (2024)	16	Gastric	ChatGPT-3.5	Tiered recommendation concordance	75%	–	Low (45%)
	19	Esophageal			63.2%	–	
	31	PDAC			64.5%	–	
	17	CCC			64.7%	–	
	10	HCC			60%	–	
Kaiser et al. (2025)	171	Prostate	ChatGPT-4	Binary concordance	Overall 93%	–	Moderate (73%)
			Claude-3-Opus				
Schmidl et al. (2024a)	20	H&N SCC	ChatGPT-3.5	Likert-scale concordance	4.3	–	Moderate (64%)
			ChatGPT-4.0		4.45	–	
Schmidl et al. (2024b)	50	H&N SCC	ChatGPT-4.0	Likert-scale concordance	4.5	–	Low (41%)
			Claude-3- Opus		4.4	–	
Schmidl et al. (2024c)	100	H&N SCC	ChatGPT-4.0	Likert-scale concordance	4.65	–	Low (45%)
			ChatGPT-4o		4.73	–	
Buhr et al. (2025)	25	ENT	ChatGPT-4o	Likert-scale concordance	84%	–	Moderate (55%)
			Llama-3		92%	–	
Ebner et al. (2024)	10	Cervical	ChatGPT-3.5	Likert-scale concordance	72.5%	–	Low (45%)
Haemmerli et al. (2023)	10	Glioma	ChatGPT 3.5	Likert-scale concordance	Moderate	–	Moderate (55%)

BC-SLM, breast cancer-small language model; PDAC, pancreatic ductal adenocarcinoma; CCC, cholangiocarcinoma; HCC, hepatocellular carcinoma; H&N, head and neck; SCC, squamous cell carcinoma; ENT, ear, nose, and throat.

Several studies employed five-point Likert scales to quantify agreement between LLM-generated recommendations and MDT decisions. In colorectal cancer, Horesh et al. reported a mean concordance score of 4.08, indicating high alignment (Horesh et al., 2025). Comparative evaluations across breast and head and neck cancers demonstrated that ChatGPT-4 consistently outperformed earlier models and contemporary alternatives, including Google Bard, Anthropic Claude, and Meta LLaMA (Zhao et al., 2020; Griewing et al., 2024a,b; Li et al., 2024). Collectively, these findings reflect the rapid evolution of LLM capabilities (Tian et al., 2020; Kim et al., 2020). While performance in structured, guideline-aligned scenarios is approaching that of traditional rule-based CDSSs, reliability in complex, context-sensitive cases remain uncertain and warrants further prospective validation.

#### 3.3.5 Other systems

A small number of studies evaluated CDSSs that did not fall into the preceding categories (Table 6). These systems primarily used supervised ML models trained on retrospective clinical datasets to predict treatment selection. In breast cancer, such models demonstrated high discriminatory performance for adjuvant treatment decisions, achieving excellent AUC values for chemotherapy, endocrine therapy, and HER2-targeted therapy (Lin et al., 2016). In esophageal cancer, predictive performance was moderate, with patient age identified as a key factor influencing the selection of neoadjuvant chemotherapy (Thavanesan et al., 2023). In hepatocellular carcinoma, model accuracy varied by treatment modality, performing better for surgical and palliative pathways than for radiotherapy or intra-arterial therapies (Daye et al., 2024). The virtual skin MDT (vSMDT), developed and applied in basal cell carcinoma, also demonstrated high accuracy across a large number of lesions (Ali et al., 2024). Collectively, these findings suggest that non-guideline-based AI systems can perform well in specific, well-defined clinical tasks. However, performance was heterogeneous, tumor-specific, and highly dependent on the characteristics of the training datasets, limiting generalizability across broader patient populations.

**Table 6:** Overview of other systems.

References	Sample size	Tumor group	Decision support	Outcome measurement	Performance of decision support	Notable findings	Quality assessment
Lin et al. (2016)	1,065	Breast	ML model	AUC	- Chemotherapy 0.940 (95% CI: 0.922–0.958) - Endocrine therapy 0.899 (95% CI: 0.880–0.918) - Trastuzumab 0.977 (95% CI: 0.955–0.999)	–	High (91%)
Thavanesan et al. (2023)	399	Esophageal	ML model	AUC	- Multinomial logistic regression 0.793 [±0.045] - Random forests 0.757 [±0.068] - Extreme gradient boost 0.740 [±0.042] - Decision tree 0.709 [±0.021]	- Age was a significant factor influencing the decision to offer surgery alone vs. neoadjuvant chemotherapy followed by surgery across models (*P* < 0.05)	High (91%)
Daye et al. (2024)	140	HCC	ML algorithm	Accuracy, AUC	- Intra-arterial therapy 0.64, 0.61 - SIRT 0.78, 0.61 - Ablation 0.69, 0.80 - Radiotherapy 0.57, 0.55 - Surgery 0.90, 0.81 - Transplant treatments 0.85, 0.88 - Chemotherapy 0.85, 0.72 - Palliative care 0.92, 0.85	–	Moderate (73%)
Ali et al. (2024)	893 (1,045 lesions)	Skin (BCC)	vSMDT	Accuracy	0.92	–	High (77%)

ML, machine learning; AUC, area under the curve; CI, confidence interval; SIRT, selective internal radiation therapy.

### 3.4 Quality assessment

Overall methodological quality assessed using the CASP checklist was predominantly moderate-to-high. Twenty-four studies were rated as high quality, 30 as moderate, and 5 as low.

## 4 Discussion

This systematic review synthesizes current evidence on the use of CDSSs in surgical oncology across multiple tumor types and international healthcare settings. Overall, CDSSs demonstrate potential to support therapeutic decision-making in MDTs, particularly by promoting guideline adherence, improving workflow efficiency, and assisting less experienced clinicians. However, their routine clinical effectiveness remains constrained by technical limitations, variation in clinical guidelines, socioeconomic and system-level factors, and patient-specific considerations.

### 4.1 Technical limitations

Early guideline-based CDSSs were built on symbolic AI, a rule-driven approach that uses predefined, human-crafted logic to generate recommendations (Eccher et al., 2014). In contrast to ML systems that can learn patterns from data and experience, symbolic AI depends on explicitly encoded rules and structured knowledge. A major limitation of these CDSSs is their reliance on continuous updating to remain clinical relevance. Timely integration of revised clinical guidelines was one of the most frequently reported barriers (Hendriks et al., 2024). This challenge is particularly problematic in oncology, where standards of care evolve rapidly with the introduction of immunotherapy and targeted treatments. Systems that fail to incorporate these changes risk generating obsolete recommendations. This issue was illustrated in Lee et al.'s study using WfO, where reimbursement changes in South Korea expanded access to biologic therapies. However, earlier versions of WfO failed to incorporate these updates and continued to recommend older chemotherapy regimens after 2013, despite widespread adoption of biologics in colorectal cancer. Consequently, concordance with MDT treatment plans declined from 64.2% to 20.3% (Lee et al., 2018). This example highlights the importance of maintaining up-to-date guideline logic within CDSSs to reflect evolving standards of care. Beyond the currency of their knowledge bases, CDSSs are inherently limited by the scope of available clinical guidelines. When patient presentations fall outside existing recommendations, these systems may be unable to generate appropriate treatment suggestions. In the Oncoguide study, Keikes et al. (2021) reported that 92% of discordant cases were attributable to the absence of relevant guideline coverage. These findings underscore the continued importance of human MDT input in interpreting and managing non-standard or complex cases for which predefined pathways or guidelines do not exist.

Furthermore, the performance of these CDSSs is influenced by the quality and completeness of clinical input data. Missing or incomplete variables, such as tumor staging, histopathological details or biomarker status, can hinder the generation of treatment recommendations or lead to suboptimal outputs. In the Oncoguide series, for example, 16.6% of cases were excluded from evaluation due to insufficient clinical information (Ebben et al., 2022). Completeness of biomarker data further affected performance. In lung cancer, concordance was significantly lower when EGFR mutation testing was unavailable, while in breast cancer, incorporation of genomic profiling improved alignment with MDT decisions (Liu et al., 2018; You et al., 2020). Unlike human MDTs, which can verify missing data or consult patient records during case discussions, CDSSs lack the capacity for real-time clarification, increasing the likelihood of absent, inaccurate, or misleading outputs. As such, successful integration of these systems into MDT workflows will require the establishment of a consistent and comprehensive minimum dataset to ensure that inputs meet the necessary threshold for reliable decision support.

### 4.2 Large language model-specific limitations

Recent CDSS research has increasingly focused on LLMs, particularly following the widespread commercial availability of systems such as ChatGPT. Emerging evidence suggests that LLMs may have potential as supportive tools within MDT workflows; however, several important limitations remain.

In a retrospective analysis of 138 soft tissue sarcoma cases discussed at an MDT, Dehdab et al. evaluated the performance of ChatGPT-4o against MDT recommendations (Dehdab et al., 2026). Overall, the model generated clinically relevant and personalized treatment recommendations with substantial expert-rated agreement. However, the study also identified several clinically important limitations, including hallucinations, confabulations, and discrepancies in treatment sequencing and chemotherapy selection when compared with expert MDT recommendations. Hallucinations and confabulations refer to the generation of plausible-sounding but inaccurate or fabricated information by AI systems. These findings likely reflect a fundamental limitation of current LLMs, which generate responses through probabilistic language associations rather than genuine clinical reasoning or understanding. Consequently, LLMs may produce outputs that appear plausible yet are clinically inaccurate, particularly in situations involving uncertainty or incomplete clinical information (Griewing et al., 2023).

Another important consideration is the influence of prompting methodology on LLM performance, with prompt engineering emerging as a major determinant of output quality, reproducibility, and clinical applicability. Several studies demonstrated that iterative refinement of structured prompts was necessary to optimize guideline-concordant recommendations (Schmidl et al., 2024a,b,c). Zabaleta et al. (2025) further showed that concordance could be improved through incorporation of clinical practice guidelines within prompts, while inclusion of detailed patient-specific variables enabled recommendations to be more effectively tailored to individual clinical scenarios. These findings highlight the dependence of current LLM performance on prompt quality, which may limit reproducibility and standardization across studies and real-world clinical implementation. Therefore, future studies evaluating concordance between LLMs and MDT recommendations should provide transparent reporting of prompt design, refinement strategies, and model parameters to facilitate meaningful comparison across studies. Together, these findings suggest that although LLMs hold promise as adjunctive tools within MDT workflows, important limitations persist, including susceptibility to hallucinations, dependence on prompt quality, and the absence of true clinical reasoning. Addressing these concerns will likely require standardized prompting frameworks, prospective clinical validation, and robust governance structures to ensure appropriate human oversight.

### 4.3 Guideline variation and socioeconomic system-level constraints

Beyond technical considerations, the effectiveness of CDSSs is influenced by variation in clinical guidelines, socioeconomic factors, and local healthcare systems. Systems developed within a specific regulatory and clinical context may therefore show reduced performance when applied internationally. This has been most evident for the commercial WfO platform, which was developed using NCCN guidelines and US drug approvals and has frequently demonstrated lower concordance in non-US settings, particularly in East Asian cohorts where clinical guidance, drug availability, and reimbursement models differ. As a result, differences in national guidelines and access to approved therapies represent a major source of discordance.

This effect is particularly evident in China, where oncology practice is guided by the Chinese Society of Clinical Oncology (CSCO) recommendations, which diverge from NCCN guidelines in several key areas. Multiple Chinese studies have reported that WfO frequently recommended NCCN-aligned therapies that were not approved, routinely available, or consistent with national practice under CSCO guidance, thereby limiting clinical applicability (Xu et al., 2019; You et al., 2020). For example, in You et al. (2020) study of metastatic non-small cell lung cancer, discordance reflected both differences between CSCO and NCCN guidelines and limited access to WfO-recommended agents such as osimertinib and bevacizumab. Similarly, Zhao et al. (2020) reported that 40.5% of patients with metastatic breast cancer were unable to access recommended CDK4/6 inhibitors because these therapies had not yet been approved by national regulatory authorities. At a regional level, higher discordance rates in cervical cancer were observed in rural areas of China, potentially reflecting reduced access to radiotherapy or targeted therapies (Zou et al., 2020).

In addition to regulatory and guideline variation, economic and reimbursement constraints further limit the feasibility of CDSS-guided recommendations in routine clinical practice. In South Korea, Choi et al. (2019) found that discordance in gastric cancer cases was largely driven by biologic therapies not covered by national insurance, leading some patients to decline WfO-recommended treatments. In China, Zhao et al. (2020) similarly reported that nearly 20% of patients declined adjuvant targeted therapy because of reimbursement limitations. Comparable cost-related barriers were also observed for high-cost prostate cancer therapies, such as enzalutamide (Park et al., 2023). Taken together, these findings suggest that discordance between CDSS recommendations and MDT decisions frequently reflects systemic healthcare constraints rather than fundamental clinical disagreement. To improve real-world applicability and clinical utility, CDSSs must be localized to regional clinical guidelines, therapeutic availability, and reimbursement frameworks.

### 4.4 Patient-specific factors

Clinical contextual factors, including individual patient characteristics such as age and performance status, were consistently associated with concordance between CDSS recommendations and MDT decisions (Somashekhar et al., 2018; Pan et al., 2019). Older or frail patients frequently receive less aggressive treatment than standard guidelines recommend, reflecting clinical judgement that balances comorbidities, functional reserve, and quality-of-life considerations. Accordingly, multiple studies reported lower concordance in elderly or comorbid cohorts, as clinicians appropriately de-escalated therapy in these populations (Yu et al., 2021; Ural et al., 2023; Park and Chae, 2024). In contrast, younger and fitter patients were more likely to receive guideline-concordant care (Xu et al., 2024). These limitations were most apparent in clinical gray areas, where standardized guidance may not apply and individualized decision-making is required. For example, a decision tree model for skin cancer achieved only 45.1% concordance with MDT decisions because it could not account for patient age, comorbidities, or lesion-specific factors (Andrew et al., 2022). Similarly, in thyroid cancer, WfO frequently recommended total thyroidectomy, whereas MDTs opted for lobectomy based on individualized risk profiles (Yun et al., 2021). Early LLM-based CDSSs demonstrated comparable shortcomings, often generating generic recommendations that failed to incorporate contextual nuance (Xu et al., 2024; Lukac et al., 2023; Griewing et al., 2024a). Finally, patient preferences frequently override CDSS-generated recommendations. Several studies reported discordance when patients opted for clinical trial participation or experimental treatments, with trial enrolment identified as a leading cause of discordance in one Oncoguide evaluation (Ebben et al., 2022). Current CDSSs remain limited in their ability to capture subjective factors such as patient values, lifestyle priorities, and individual treatment goals, all of which strongly influence real-world clinical decision-making.

Beyond patient-level factors, tumor-specific characteristics also influenced CDSS concordance. Disease stage in particular affected concordance, although findings across studies were heterogeneous. Higher concordance was reported in some advanced disease cohorts, lower concordance in others, and no significant stage-related differences were observed in one lung and one breast cancer study (Somashekhar et al., 2018; Zhou et al., 2019; Pan et al., 2019; Liu et al., 2023). In a meta-analysis by Oehring et al. (2023) concordance rates were similar for early-stage (72.7%) and advanced-stage disease (73.4%) across multiple tumor groups. For breast cancer specifically, concordance increased from 72.8% in early-stage disease to 84.1% in advanced disease. While the underlying mechanisms were not formally explored, higher concordance in advanced disease may reflect more clearly defined, guideline-driven treatment pathways. In contrast, early-stage disease often permits greater clinical discretion and incorporation of patient preferences, such as surveillance strategies in indolent prostate cancer or breast-conserving approaches, which may reduce alignment with algorithm-based recommendations. Conversely, in advanced disease, some patients may pursue experimental or novel therapies not yet incorporated into CDSS knowledge bases, also contributing to discordance (Ebben et al., 2022). Taken together, these findings indicate that CDSSs are most effective when applied to cases that closely align with established clinical guidelines. Patients requiring nuanced, individualized care, particularly those who are frail, comorbid, or preference-sensitive, may be less well-served by algorithmic recommendations alone. Accordingly, CDSSs should be positioned as adjuncts to, rather than replacements for, clinical judgement within MDT decision-making.

### 4.5 Impact on MDT processes

Despite the limitations outlined above, CDSSs offer several potential benefits for MDT practice. Reported advantages include improved adherence to clinical guidelines, enhanced identification of clinical trial eligibility, improved efficiency through the triage of routine cases, and support for less experienced clinicians. Among the reported benefits, improved adherence to clinical guidelines was one of the earliest and most consistently reported benefits following CDSS implementation. For example, the introduction of OncoDoc2 increased guideline adherence in breast cancer MDTs from 79.2% to 93.4%, while WfO modestly improved alignment with recommended care in colorectal cancer (Séroussi et al., 2007; Lee et al., 2018). In some instances, discordance reflected MDT deviation from established guidelines rather than CDSS error, suggesting that these systems may function as a “guideline safeguard.” By embedding guideline-based recommendations into MDT workflows, CDSSs may help reduce cognitive bias, promote consistency, and minimize unwarranted variation in clinical decision-making. Beyond reinforcing guideline adherence, CDSSs may also add value by supporting standardized cases while simultaneously broadening consideration of alternative treatment options in more complex clinical scenarios. This is further illustrated by their potential role in identifying clinical trial eligibility; for example, the MATE system identified substantially more eligible patients than standard MDT review, highlighting its capacity to expand access to novel therapies and improve trial recruitment (Patkar et al., 2012). Together, these findings suggest that CDSSs may contribute both to standardized, guideline-aligned care and to more comprehensive exploration of therapeutic options in complex cases.

Moreover, several studies reported potential gains in MDT efficiency through the triage of routine or low-risk cases using CDSSs. Certain models were able to reliably identify patients suitable for streamlined pathways, potentially allowing MDTs to focus discussion time on more complex cases (NHS England, 2020; Patkar et al., 2012; Andrew et al., 2022). Such approaches may facilitate more effective prioritization of MDT agendas, ensuring that cases requiring nuanced multidisciplinary input receive adequate time and attention while preserving decision quality for more straightforward presentations.

An additional advantage of guideline-based CDSSs is their transparency. Recommendations can typically be traced to specific evidence or guideline sources, allowing MDTs to interrogate and validate proposed management plans. This feature may be particularly beneficial for less experienced clinicians. McNamara et al. (2019) demonstrated that oncologists with limited breast cancer subspecialty expertise achieved greater alignment with expert decisions when using WfO, while specialist decision-making remained largely unchanged. This suggests that CDSSs may function as a “virtual expert,” helping to mitigate knowledge gaps in resource-limited settings or in circumstances where full MDT quoracy is difficult to achieve.

However, these potential benefits must be balanced against the risk of automation bias. Automation bias refers to the tendency to favor recommendations generated by automated decision-making systems while disregarding contradictory information from non-automated sources, even when the latter is correct. Excessive reliance on AI recommendations may lead clinicians to accept incorrect outputs without sufficient critical evaluation. Kücking et al. (2024) investigated automation bias in a study in which healthcare professionals diagnosed wound-healing complications using an AI-based CDSS that provided diagnostic suggestions. In half of the cases, the AI system intentionally generated incorrect recommendations. The study demonstrated that clinicians with greater expertise, experience, and clinical competency were less susceptible to automation bias and were therefore less likely to accept false AI recommendations. Within this context, CDSS tools cannot replace clinical expertise or intensive professional training; rather, their safe and effective implementation depends upon it. Current AI-based CDSSs should therefore be implemented as adjuncts to clinical decision-making rather than autonomous systems, with a “human-in-the-loop” approach maintained to provide expert oversight and mitigate the risks of automation bias.

Despite these process-related benefits, evidence linking CDSS use to patient outcomes remains limited. Only a small number of studies examined concordance in relation to clinical outcomes, primarily survival. Mao et al. (2020) reported 66.9% concordance in colorectal cancer, with improved overall survival observed in concordant cases, while Aikemu et al. (2021) reported 91% concordance with similar survival benefits. In gastric cancer, Tian et al. (2020) demonstrated longer mean survival in concordant compared with discordant cases (30 months vs. 16.4 months). Across several analyses, non-concordance was identified as an independent adverse prognostic factor (Tian et al., 2020; Mao et al., 2020). However, these associations should be interpreted cautiously, as they may be influenced by confounding variables. Lower concordance was more frequently observed in patients with advanced-stage disease, poorer performance status, or greater comorbidity burden, suggesting that observed survival differences may partly reflect baseline disease severity and patient fitness rather than the direct effect of CDSS-guided decision-making (Kim et al., 2019a; Mao et al., 2020). Overall, while several potential benefits of CDSSs for MDT processes are evident, most studies in this review focused on concordance with MDT decisions or clinical guidelines rather than direct patient outcomes. Concordance alone is a limited surrogate for clinical value, as MDT decisions are inherently context-dependent and variable, and agreement does not necessarily imply correctness or improved clinical outcomes.

### 4.6 Limitations of this review

The evidence base included in this review was predominantly observational. Most studies consisted of retrospective cohort analyses comparing CDSS recommendations with MDT decisions, with some incorporating blinded reviewers to minimize bias. Although seven large studies (each including over 1,000 cases) provided relatively robust data, many were small (22 studies included ≤ 100 patients) and conducted at single centers, limiting generalisability. Furthermore, only a minority of studies evaluated patient outcomes, making it difficult to determine how CDSS recommendations translate into real-world clinical outcomes (Hikal et al., 2023; Daye et al., 2024).

Considerable heterogeneity was also evident across studies in terms of patient populations, study design, and performance outcome reporting. This inconsistency in concordance reporting was particularly apparent among the WfO studies. Most studies reported concordance using tiered recommendation categories (“overall” concordance, incorporating both “recommended” and “for consideration” recommendations, alongside separate “recommended,” “for consideration,” and “not recommended” categories). In contrast, several studies used binary concordance (“concordant” and “non-concordant”), generally defined as agreement with both the “recommended” and “for consideration” categories (Liu et al., 2023; Park et al., 2023). In some studies, however, binary concordance was restricted to the “recommended” category alone, excluding “for consideration” (where applicable, this has been specified in Table 4). Consequently, studies reporting low concordance within the “recommended” category may have demonstrated substantially higher overall agreement when “for consideration” options were included. Outcome reporting also differed among non-WfO studies. Several LLM-based studies assessed concordance using expert-rated Likert scales rather than binary concordance, with clinicians evaluating the degree of alignment between CDSS-generated recommendations and MDT decisions (Table 5) (Schmidl et al., 2024a,c; Horesh et al., 2025). This heterogeneity in outcome reporting complicates cross-study comparisons and limits interpretation of CDSS performance across different systems.

Furthermore, publication bias remains a potential concern, as studies reporting low concordance, particularly those involving commercial systems such as WfO, may be underrepresented in the literature. Overall, the included studies were of moderate quality. Although the use of real-world clinical data represents a notable strength, the predominance of retrospective, single-center studies and the lack of prospective, randomized, or multi-center trials limit the overall strength and generalisability of the evidence.

### 4.7 Future research

AI-based CDSSs hold substantial promise for supporting MDT decision-making but should complement, rather than replace, clinical expertise. Future research should therefore evaluate the impact of CDSSs on clinically meaningful endpoints, including changes in clinical decision-making, treatment sequencing, and patient outcomes such as complication rates, recurrence, and survival. In parallel, real-world implementation studies examining feasibility, human factors, and downstream clinical impact are needed to better define the role of CDSSs within MDT workflows. Beyond clinical outcomes, future research should also explore stakeholder perspectives, including patient and clinician attitudes toward AI-assisted treatment planning, and address issues related to responsible integration and accountability when AI-generated recommendations are incorrect. Ongoing development should prioritize large prospective studies to robustly assess real-world impact on both patient outcomes and MDT performance.

## 5 Conclusion

This systematic review examined the current landscape of AI-based CDSSs designed to support MDT therapeutic decision-making. Across 59 included studies, substantial variability in performance was identified, reflecting technical limitations, socioeconomic and healthcare system constraints, and patient- and tumor-specific factors. Despite these challenges, several potential benefits were reported. These included improved adherence to clinical guidelines, greater consistency in treatment recommendations, enhanced identification of patients eligible for clinical trials, support for less experienced clinicians, and opportunities to streamline MDT workflows through the triage of routine or low-risk cases. However, effective implementation requires careful localization to regional clinical guidelines, drug availability, reimbursement structures, and healthcare infrastructure, alongside timely updates to reflect evolving evidence and regulatory approvals. Importantly, AI-based CDSSs should be positioned as adjuncts to clinical decision-making, with appropriate human oversight rather than as autonomous decision-making tools. Future research should prioritize real-world implementation studies that extend beyond technical performance and concordance metrics to evaluate their impact on clinical decision-making, treatment sequencing, and patient-centered outcomes, including complications, recurrence, and survival. In parallel, further investigation into feasibility, usability, human factors, and integration within MDT workflows is needed to more clearly define the role of CDSSs in contemporary oncology practice.

## Data Availability

The original contributions presented in the study are included in the article/supplementary material, further inquiries can be directed to the corresponding authors.
